# Radio-opacity of the Bones of Commonly Consumed Fish from the Red Sea

**DOI:** 10.7759/cureus.6473

**Published:** 2019-12-26

**Authors:** Abdullah K AlBathi, Saeed S Shaaban, Faisal Alshadadi, Bader Alsheikh, Basim Althinayyan, Khalid Khashoggi, Mazin Merdad

**Affiliations:** 1 Department of Otolaryngology - Head and Neck Surgery, King Abdulaziz University Hospital, Jeddah, SAU; 2 Department of Medicine, King Abdulaziz University Hospital, Jeddah, SAU; 3 Department of Radiology, King Abdulaziz University Hospital, Jeddah, SAU

**Keywords:** fish bones, radio-opacity, foreign body, foreign body impaction, x-ray, emergency, red sea, fish

## Abstract

Introduction

Foreign body (FB) ingestion is one of the most common complaints presenting at an emergency department (ED), with fish bone impaction being a frequent cause of presentation. Fish bones might be challenging to identify on routine radiography and ED physicians are often left in a state of uneasiness owing to the fear of complications occurring if the fish bone is not removed.

Objective

This study aimed to establish the factors affecting the radio-opacity of fish bones on X-ray.

Materials and methods

The study involved the top three fish species consumed on Saudi Arabia’s western coast. Fish bones from three specimens of each species were radiographically examined by hand-picking bones from different parts of the fish, with particular attention paid to bones that are difficult to spot. Bones were then arranged beside each other, and radiographs were taken for comparison. Inter-species and intra-species radio-opacity variation was tested. Further, the weight of each fish and method of cooking (baked vs. fried) were tested for their effect on radio-opacity.

Results

No significant difference in radio-opacity was found among and between different species, and the method of cooking did not alter the radio-opacity of fish bones. Significant differences in radio-opacity were noted with the difference in the diameter and size of the fish bones, which tended to be less radio-opaque in smaller-sized fish, regardless of the species.

Conclusion

The exact fish species and method of cooking did not alter the fish bone density on an X-ray. The size of the fish and the size of the fish bone are better predictors of higher fish bone density.

## Introduction

Foreign body (FB) ingestion is a common complaint of patients presenting to the emergency department (ED) [1]. The most frequently impacted FBs in the upper aerodigestive tract include bones (fish and other), dentures, coins, food boluses, and seeds [1-2]. The frequency of FB ingestion varies between age groups. The highest prevalence is seen in the pediatric group (commonly coins and seeds) and middle-aged adults (commonly fish bones and dentures) [3-4].

Fish bones are the most commonly ingested FBs across Asia, the Mediterranean, and other coastal regions [3,5-7]. Most of the accidentally ingested fish bones harmlessly pass through the digestive system; however, some can get impacted within the gastrointestinal tract, most commonly in the oropharynx or esophagus [2-3,8-11]. Fish bones have a higher chance of causing bleeding and/or perforations in the esophagus than other FBs [3].

Given the variety of their shapes, sizes, and radio-opacities, identifying fish bones on radiographs is a common challenge facing ED physicians. Previously published studies on fish bones, albeit limited in number and sample size, found that the radio-opacity of fish bones could vary according to the type of fish, its size, and the method of cooking [3,5-6]. The type of fish consumed, however, cannot be recalled or identified by most patients [12].

In our study, we examined the commonly consumed local fish found along the western coast of Saudi Arabia and aimed to identify the ease of detecting different fish bones on X-ray imaging. We assessed the presence of inter-species or intra-species variation in terms of bone radio-opacity. Further, we assessed if the fish weight and method of cooking have an effect on the radio-opacity of its bones.

## Materials and methods

This research project has obtained ethical approval by the institutional review board at King Abdulaziz University, Faculty of Medicine.

The fish commonly consumed locally along the western coast of Saudi Arabia include “Najil” or Coral Trout (Plectropomus leopardus); “ Hamour” or Greasy Grouper (Epinephelus tauvina); “Hareed” or Rusty Parrotfish (Scarus ferrugineus); and “Shaour” or Orange-striped Emperor (Lethrinus obsoletus). We selected fish that are of the average weight of that particular species (according to their availability at the fish market), ranging from 0.5 kg to 1 kg.

All radiographic images were reviewed by a radiology consultant and an ear, nose, and throat (ENT) consultant at King Abdulaziz University Hospital. There are no objective methods of assessing the radio-opacity of fish bones, and the degree of radio-opacity in our study depended on the subjective assessment of two expert consultants.

The fish bones were radiographed to answer three questions: Is there a difference in fish bone radio-opacity between different fish types? Is there a difference in fish bone radio-opacity based on the cooking method (baking vs. frying)? Is there a difference in fish bone radio-opacity based on the size (total weight) of the fish?

To test the inter-species variation and effect of cooking method on fish bone radio-opacity, two fish from each species were cooked using a different method (frying and baking). Each fish was cut in half down the middle; one half (cranial part) was fried and the other half (caudal part) was baked. The bones were hand-picked from different parts of the fish, with attention to hand-pick ones that are difficult to spot. Based on their average weights, we selected two fresh fish - one large and one small - from each species, to test the effect of fish size on bone radio-opacity.

## Results

Is there a difference in fish bone radio-opacity between different fish species?

**Figure 1:**
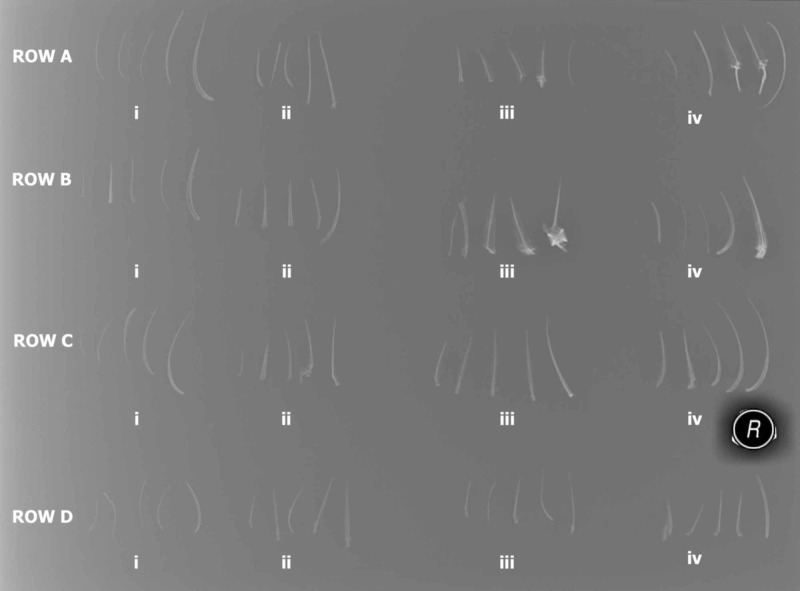
The radio-opacity on fish bones according to the fish species and cooking method Row A: Lethrinus obsoletus (Shaour), (i, ii) cooked by frying; (iii, iv) cooked by baking Row B: Scarus ferrugineus (Hareed), (i, ii) cooked by frying; (iii, iv) cooked by baking Row C: Plectropomus leopardus (Najil), (i, ii) cooked by frying; (iii, iv) cooked by baking Row D: Epinephelus tauvina (Hamour), (i, ii) cooked by frying; (iii, iv) cooked by baking (i, iii) are from the same fish; (ii, iv) are from the same fish

Our first study set demonstrated no remarkable difference in the radio-opacity of fish bones within and between species. The fish bones were arranged on an X-ray plate, as shown in Figure 1.

Is there a difference in fish bone radio-opacity based on the cooking method?

The method of cooking did not seem to affect the radio-opacity of the bones, as can be seen in Figure 1. Baking or frying fish did not have an effect on the radio-opacity of fish bones.

Is there a difference in fish bone radio-opacity based on the size (total weight) of the fish?

**Figure 2:**
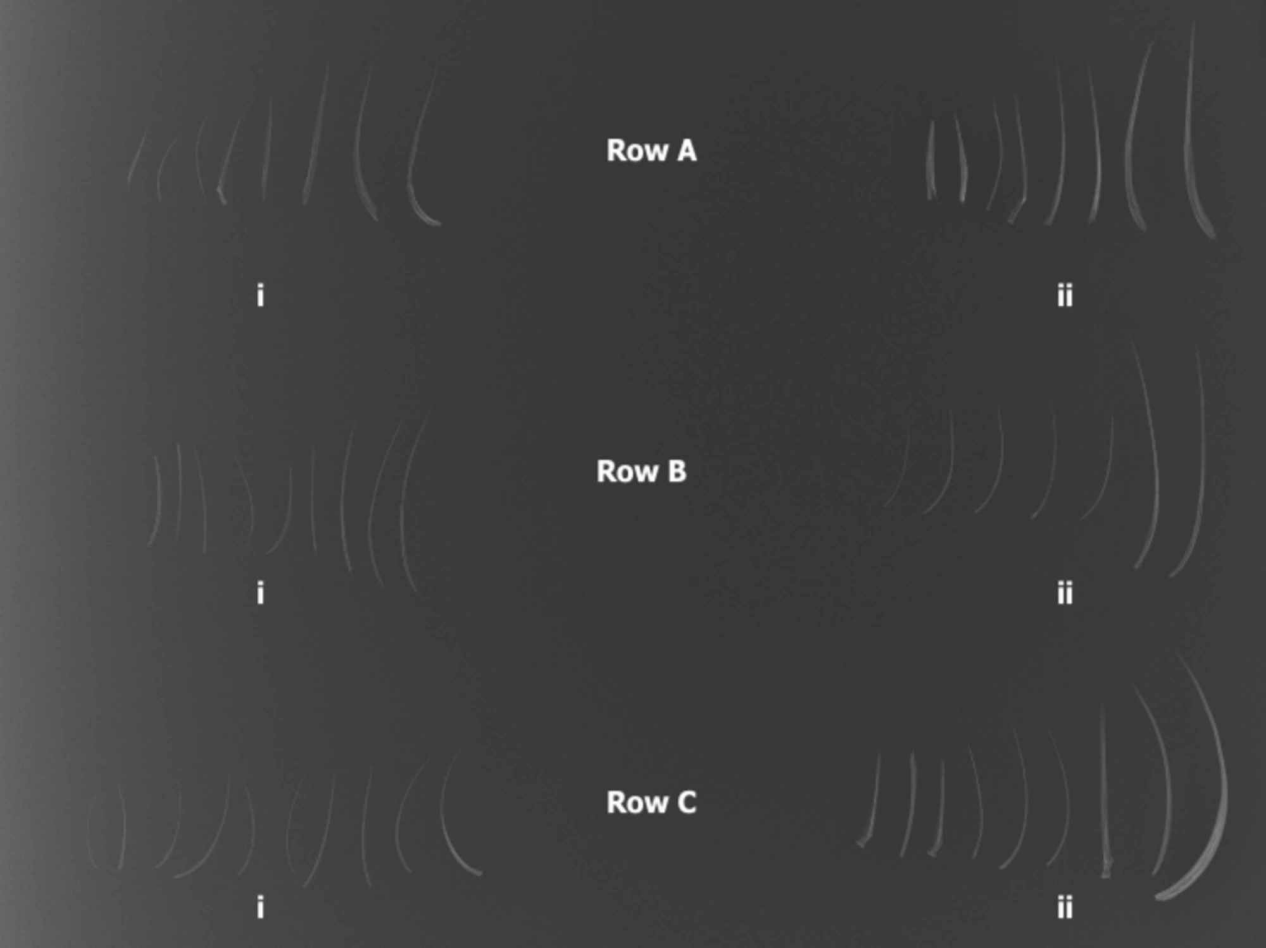
The radio-opacity on fish bones according to the fish size (total weight) Row A: Lethrinus obsoletus (Shaour), (i) 0.400 kg; (ii) 1.380 kg Row B: Scarus ferrugineus (Hareed), (i) 0.800 kg; (ii) 1.620 kg Row C: Epinephelus tauvina (Hamour), (i) 0.370 kg; (ii) 1.960 kg All fish have been baked.

In the second study set, all fish samples were baked, as the first set showed minimum variability in radio-opacity between the two methods of preparation. The fish bones were arranged on the X-ray plate sequentially by size, as shown in Figure 2.

Among the Red Sea fish (Greasy Grouper (Epinephelus tauvina), Coral Trout (Plectropomus leopardus), Rusty Parrotfish (Scarus ferrugineus], and Emperor (Lethrinus obsoletus)) the only significant differences noted in radio-opacity were due to the diameter and size of the fish bone. Overall, larger fish had larger bones and were generally more radio-opaque.

## Discussion

No study has previously tried to assess the radio-opacity of fish bones that are commonly consumed in the western region of Saudi Arabia. The utility of using radiographic tools to assess the presence of fish bone FBs continues to be a controversial topic.

Our results are consistent with a study done in Malaysia, which found that the length and thickness of a fish bone influence its visualization on radiography [13]. Furthermore, our study found that the method of cooking - frying or baking - did not affect the bones’ opacity level. This corresponds to a study by Lue et al., which reported similar findings. There is insufficient evidence in the literature to support that the method of cooking affects fish bone radio-opacity [14].

The bones of different species of the Red Sea fish that were included in our study did not differ in radio-opacity. Other studies, however, namely, by Lue et al. and Ell et al., reported that different species of fish differ in the optical density of fish bones. Lue et al. studied 14 species of fish while Ell et al. studied 15 types of fish [14-15]. We were not able to elicit such a difference in commonly consumed Red Sea fish.

Since patients usually present to the ED after the ingestion of small bones that were neither visible in their food nor felt in their mouths, we believe that there is a possibility that most of the bones ingested are unlikely to be visualized using radiographic tools. Our results show that bones of larger fish are easier to detect on X-rays than the bones of smaller fish. Thus, noting the size of the fish consumed by patients presenting at the emergency room might be an important fact to elicit. A limitation of our study is the lack of documentation of the size of fish consumed by patients presenting to the ED with complaints of fish bone ingestion. If possible, this should be recorded in the patients’ histories, which would help in determining the likelihood of X-ray detection.

Future studies of the radio-opacity of fish bones may include a higher number of fish and fish from different species from the Red Sea. Additionally, a retrospective or prospective study that examines the radiographs of patients presenting to the ER with fish bone ingestion could be of more practical and clinical use. Further, establishing the use of an objective assessment tool in defining the radio-opacity or density of fish bones in X-ray images, such as the Hounsfield scale that is currently used in computed tomography (CT) images, could be a valuable tool in quantifying our and future data sets.

## Conclusions

In conclusion, the size and diameter of the fish bone are the only important factors affecting the radiolucency or radio-opacity of commonly consumed Red Sea fish bones on X-ray images. The study also determined that the exact fish species and method of preparation did not alter the fish bone radiolucency on the X-ray images.
